# Boron doped silver-copper alloy nanoparticle targeting intracellular *S*. *aureus* in bone cells

**DOI:** 10.1371/journal.pone.0231276

**Published:** 2020-04-10

**Authors:** Tahir Abdulrehman, Shahnaz Qadri, Sini Skariah, Ali Sultan, Said Mansour, Jamil Azzi, Yousef Haik

**Affiliations:** 1 College of Health and Life Sciences, Hamad Bin Khalifa University, Doha, Qatar; 2 College of Science and Engineering, Hamad Bin Khalifa University, Doha, Qatar; 3 Weil Cornell Medicine-Qatar, Education City, Doha, Qatar; 4 Qatar Environment and Energy Research Institute (QEERI), Hamad Bin Khalifa University, Doha, Qatar; 5 Brigham and Women’s Hospital, Harvard Medical School, Boston, United States of America; VIT University, INDIA

## Abstract

**Objectives:**

Alloyed metallic nanoparticles of silver and copper are effective against intracellular infection. However, systemic toxicity may arise due to the non-specific delivery of the nanoparticles. In addressing the issue, this study deals with the targeting of silver-copper-boron (ACB) nanoparticles to infected osteoblasts, which could decrease systemic toxicity and form the basis of targeting specific markers expressed in bone infections.

**Methods:**

ACB nanoparticles were synthesized and conjugated to the Cadherin-11 antibody (OBAb). The effect of targeting nanoparticles against extracellular and intracellular *S*. *aureus* was determined by enumeration of bacterial growth. The binding of the targeting nanoparticles to infected osteoblasts as well as the visualization of live/dead bacteria due to treatment was carried out using fluorescence microscopy. MTT assay was used to determine the viability of osteoblasts with different concentrations of the nanoparticles.

**Results:**

The ACB nanoparticles conjugated to OBAb (ACB-OBAb) were effective against extracellular *S*. *aureus*. The ACB-OBAb nanoparticles showed a 1.32 log reduction of intracellular *S*. *aureus* at a concentration of 1mg/L. The ACB-OBAb nanoparticles were able to bind to the infected osteoblast and showed toxicity to osteoblasts at levels ≥20mg/L. Also, the percentage of silver, copper, and boron in the nanoparticles determined the effectiveness of their antibacterial activity.

**Conclusion:**

The ACB-OBAb nanoparticles were able to target the osteoblasts and demonstrated significant antibacterial activity against intracellular *S*. *aureus*. Targeting shows promise as a strategy to target specific markers expressed on infected osteoblasts for efficient nanoparticle delivery, and further animal studies are recommended to test its efficacy *in vivo*.

## Introduction

One of the most commonly associated pathogens with bone infection is *Staphylococcus aureus* (*S*. *aureus*), and such infections clinically manifest in the form of osteomyelitis, orthopedic prosthetic infections, and septic bacterial arthritis [1–6]. *S*. *aureus* infections result in foot ulcers, which are common in immune-compromised diabetic patients due to hyperglycemia, and chronic osteomyelitis may result in amputation of associated limbs [7,8]. Besides bone infections, *S*. *aureus* can also cause gastrointestinal, respiratory, skin, blood-stream, and heart (endocarditis) infections [9–11]. Such infections have resulted in increased health care costs as well as high mortality and morbidity [12–14]. The front-line treatment strategy against *S*. *aureus* associated bone infections includes antibiotics and surgery, but in recent years recurrence of infections has been witnessed due to the inefficacy of the current treatment strategies [15,16]. Many causes result in such failures, with a major contributing factor being the indiscriminate use of antibiotics leading to the emergence of multidrug-resistant *S*. *aureus strains*, which are resistant to potent drugs like methicillin and vancomycin [17,18]. *S*. *aureus* can survive intracellularly and evade host immune response mechanisms [19–22]. The low intracellular bioavailability and cellular permeability of antibiotics also promote the antibiotic resistance of intracellular pathogens [23,24]. The formation of a low metabolically active small colony variant (SCV) *S*. *aureus* phenotype enables it to be less susceptible to antibiotics [25,26]. *S*. *aureus* can also form biofilms that promote the survival and persistence of the pathogen in harsh conditions and also provide antibiotic resistance by limiting antibiotic penetration [27,28]. Thus, there is an urgent need for newer strategies/drugs that could effectively eradicate biofilms and intracellular pathogens as well as counter the multidrug resistance of *S*. *aureus*.

Metallic nanoparticles (size range 1 to 100 nm) of silver, copper, and their alloys are known to be antimicrobial against a wide range of infectious pathogens (bacteria, fungi, and viruses) and are promising as antimicrobial agents against *S*. *aureus* infections [29–34]. Also, alloy metallic nanomaterials with the combination of silver and copper (Ag-Cu) are found to be more potent than individual silver (Ag) or copper (Cu) nanoparticles [29,35–37]. The enhanced antimicrobial effect of bimetallic alloy Ag-Cu may be due to the synergistic release of Ag^+^ and Cu^2+^ ions that cause DNA damage in bacteria [38,39]. Metallic nanoparticles have a multimodal mechanism of antibacterial action, which also includes bacterial cell membrane damage, intracellular damage, and induction of oxidative stress [40–43]. The metallic ions can also penetrate the mammalian cells and thereby induce intracellular antimicrobial activity [44–46]. Similar to metallic nanoparticles, compounds containing the metalloid boron also exhibit antimicrobial properties [47–49]. Also, boron and its compounds have an anticorrosive effect on the copper metal, preventing the formation of copper oxides [50,51]. Metallic copper nanoparticles exhibit enhanced release of copper ions in solutions when compared to the copper oxides [52]. In our previous studies, it has been shown that tri-elemental Silver-copper-boron (ACB) nanoparticles are effective against extracellular and intracellular *S*. *aureus* both in vitro and *in vivo* [38,53], however, an overdose could result in hepatotoxicity [54].

Targeting the therapeutic agent to the infection site may reduce systemic toxicity and dosage [55–57]. Surface functionalization and conjugation to specific targeting ligands enable the effective delivery of the therapeutic agents to the infected site [56,58,59]. However, surface modification of metallic nanoparticles with ligands or biomolecules may affect their antibacterial property [60,61]. Cadherin-11 is the most abundant cadherin expressed by human osteoblasts [62,63] and was used as a targetable entity for our study. Many studies on the antimicrobial activity of metallic nanoparticles against *S*. *aureus* have been reported. However, there are only limited reports on the targeting of antibacterial metallic nanoparticles to the infected site. Hence, the objectives of this study were to target *S*. *aureus* infected osteoblasts using silver-copper-boron (ACB) nanoparticles, determine the efficacy of modified nanoparticles against intracellular infection, and also to determine the nanoparticle toxicity to osteoblasts. To fulfill the objectives, the antibacterial ACB nanoparticles were functionalized with carboxylic groups and conjugated to the Cadherin-11 antibody or anti-osteoblast cadherin antibody (OBAb) for targeting. The extracellular and intracellular antibacterial activity was determined by incubating the targeting nanoparticles (ACB-OBAb) with *S*. *aureus* and infected osteoblasts, respectively, followed by evaluating the optical density or CFU count. It is believed that this is the first study to report the targeting of infected osteoblasts and the treatment of intracellular infection using OBAb conjugated ACB nanoparticles.

## Materials and methods

### Materials

Silver Nitrate, Copper Acetate, Boric acid, Sodium Hydroxide (NaOH), 3-Mercaptopropionic acid (MPA), 1-Ethyl-3-(3-dimethyl aminopropyl) carbodiimide (EDC), Tryptic Soy Agar, Tryptic Soy Broth, Bovine serum albumin (BSA), Saponin, Hydrazine hydrate, Acetone, Absolute ethanol, and Sodium Citrate were purchased from Sigma-Aldrich, USA. Dulbecco's Modified Eagle Medium: Nutrient Mixture F-12 (DMEM/F12) Phenol free Culture Media, Fetal Bovine Serum, Sulfo-N-Hydroxysuccinimide, Phosphate Buffer Saline-pH 7.4 (PBS), DAPI [1mg/mL] (62248), FITC (46425), Goat anti-Mouse IgG (H+L) Cross-Adsorbed Secondary Antibody, Alexa Fluor 555 (A-21422) and Geneticin (G418 sulfate), Vybrant MTT assay kit were purchased from Thermofisher Scientific, USA. Anti-Osteoblast Cadherin Antibody [OBAb] (ab151446), rabbit anti-*S*. *aureus* antibody—FITC (ab68950), Goat Anti-Mouse IgG H&L [Alexa Fluor 488] (ab150113), and Goat Anti-Mouse IgG H&L [Alexa Fluor 594] (ab150116) were purchased from Abcam, USA.

### Synthesis of ACB nanoparticles

The ACB (Ratio—Ag: ~60%, Cu: ~30%, B: ~10%) nanoparticles were synthesized as follows: 0.6mL of 0.1M Silver nitrate, 0.3mL of 0.1M Copper Acetate and 0.1mL of 0.1M Boric acid was mixed in 3mL of deionized water and mixed vigorously for 5 min using a magnetic stirrer. This solution was then added dropwise to a solution containing 1mL of 0.1M MPA, 2mL of 0.1M NaOH, 0.1mL of 0.1M NaBH_4_ and 3mL deionized water under stirring and inert argon atmosphere for 5 min. This solution is added dropwise to a stirred solution containing 9.55mL of deionized water, 250μL of 0.5M hydrazine hydrate, and 200μL of sodium citrate. The solution was kept stirring for 4 h under an inert atmosphere. The nanoparticles formed were precipitated using acetone and centrifuged at 4000 rpm. The nanoparticles were then washed several times with absolute ethanol and centrifuged at 4000 rpm to collect the nanoparticles and finally freeze-dried the nanoparticles. A similar procedure was used to synthesize different ratios of ACB or AC and/or Copper alone nanoparticles. But the silver alone nanoparticles was prepared using the sodium borohydride method in which instead of hydrazine hydrate, 0.5M of sodium borohydride was added in the above procedure.

### Characterization of nanoparticles

The nanoparticles were suspended in deionized water and 10μL of nanoparticle was placed on a copper grid coated with holey carbon and kept in a vacuum desiccator for drying. The size and morphology of the nanoparticles were determined using Transmission Electron Microscopy, TEM (FEI, Talos F200X). The Energy Dispersive X-ray (EDX) installed on Talos F200X was utilized to determine the distribution and percentage of the elements in the nanoparticles. The Zetasizer (ZSP, Malvern, UK) was used to determine the hydrodynamic size and zeta potential of the nanoparticles.

### Functionalization (conjugation) of ACB with anti-osteoblast cadherin antibodies

To 1mg of ACB nanoparticles in 100μL of MES buffer (pH 6), 100μL of 10mg/mL of EDC in MES buffer was added mixed well and followed by the addition of 100μL of 26mg/mL Sulfo-NHS in MES buffer. The reaction was allowed to proceed for 20 min, followed by the centrifugation of activated nanoparticles in 10KDa MWCO (Nanosep, Pall, USA) at 8000 rpm. To the activated ACB, 950μL of PBS was added, followed by the addition of 50μL of 1mg/mL OBAbs for conjugation to the ACB nanoparticles. The percentage of OBAb conjugation was determined from the difference in the amount of unconjugated OBAb in the supernatant using the Bradford protein assay and measuring the absorbance at 595nm in the spectrophotometer (TECAN-SPARK). The conjugated nanoparticles were washed three times by centrifugation, stored at 4°C and used for the antimicrobial assay and further study. The ACB-OBAb and ACB were incubated with Alexa fluor 488 goat anti-mouse IgG (secondary) for 2 h at room temperature in the dark. The nanoparticles were washed with PBS by centrifugation, and the fluorescence intensity (Ex: 495nm/Em: 519nm) was measured using a plate reader (TECAN-SPARK, Austria). Fourier Transform Infrared (FTIR) was conducted on ACB and ACB-OBAb using a Nicolet iS50 FT-IR spectrophotometer within the range of 4000–500 cm^-1^ to determine the amide bonds of OBAb that get conjugated to ACB. The sample discs for FTIR measurement were prepared using KBr.

### *Staphylococcus aureus* culture

*S*. *aureus* (ATCC 29213, USA) strain was obtained from ATCC, cultured and maintained on Tryptic Soy Agar (TSA) plates and the stock bacteria were stored in glycerol at -80°C. Single *S*. *aureus* colony was inoculated into tryptic Soy broth (TSB) and cultured overnight at 37°C. The overnight S. aureus culture was sub-cultured by inoculating 0.5mL of overnight culture in 4.5mL of fresh TSB and grown till the mid-log phase corresponding to an optical density of 0.4. The bacterial cells were centrifuged at 1500 rpm for 15 min and washed with PBS. The bacterial cells were then inoculated in DMEM/F12 medium containing 10% FBS and were used further for antimicrobial assay and osteoblast invasion assay. The growth of *S*. *aureus* in DMEM/F12 has been shown in our previous studies^38^.

### FITC labeling of *S*. *aureus*

*S*. *aureus* was grown overnight at 37°C to achieve 10^8^ CFUs/mL in 5 mL tryptic soy broth. The culture was then washed in PBS (pH 7.4) and centrifuged at 2000 rpm. The pellet was resuspended in 5 mL of 100mM carbonate buffer (pH 9) containing 50 mg/L of FITC followed by 1 hincubation at room temperature. The FITC labeled *S*. *aureus* was centrifuged at 2000 rpm and washed with PBS (pH 7.4) and stored in 1% glycerol at -80°C until further use.

### Antimicrobial assay

The antimicrobial assay was conducted in a 24 well plate in which the wells were loaded with 1mL of DMEM/F12 (with 10% FBS) culture medium containing 1x10^5^ CFU/mL of *S*. *aureus*. The assay was conducted in triplicates and similar to a macro-dilution antimicrobial assay, the bacteria were treated with varying concentrations of antibody conjugated or non-conjugated ACB ranging from 0 (control) to 20 mg/L. The 24 well plate was incubated at 37°C, and the optical density was measured using a spectrophotometer (TECAN-SPARK, Austria) after 6 h of incubation.

### Intracellular antimicrobial assay

The human osteoblast (hFOB 1.19, ATCC CRL-11372) was obtained from ATCC and cultured in Phenol free DMEM/F12 media with 10% FBS and 0.3mg/L of G418 as per ATCC recommendations. 0.4 x 10^5^ Osteoblast cells were seeded into a 48 well sterile tissue culture plate and cultured overnight. The overnight cultured osteoblasts were washed 5 times with antibiotic-free DMEM/F12 culture medium and then infected with *S*. *aureus* at a ratio of 10:1 (*S*. *aureus*: Osteoblast) for 90 min at 37°C. The cells were then washed 3 times with antibiotic-free DMEM/F12 culture medium and incubated for 2 h in 1ml of DMEM/F12 medium containing 200 mg/L of gentamicin to remove the extracellular *S*. *aureus*. After eradication of extracellular infection, the osteoblast cells were washed three times with antibiotic-free medium, and the osteoblast cells were treated with 1mL culture medium containing varying concentrations of ACB-OBAb or ACB nanoparticles at 0, 2.5, 5, 10 and 20 mg/L and incubated for 24 h at 37°C. The experiment for each nanoparticle treatment concentration was carried out in a minimum of triplicates. After incubation, the osteoblasts were trypsinized, washed, and lysed using lysis buffer (0.5% Triton-X-100 in PBS) and serially diluted (10^−1^ to 10^−7^). 10 μL of each dilution was subjected to spot dilution assay in a TSA plate and incubated overnight, and the bacterial CFUs formed in each dilution were enumerated after overnight incubation.

### Fluorescent microscopy

The internalization of *S*. *aureus* in osteoblasts was visualized using fluorescent microscopy. The osteoblast cells (5 x 10^4^ cells) were seeded on D-lysine coated glass-bottom (14mm) imaging dishes (Mattek corporation) and cultured overnight at 37°C. The osteoblasts were infected with FITC labeled *S*. *aureus* at a ratio of 10:1 (S. aureus: Osteoblast) for 90 min. The infected osteoblasts were treated with gentamicin and washed with antibiotic-free DMEM/F12 media, as mentioned above in the intracellular antimicrobial assay. The infected osteoblasts were then cultured in 1mL of antibiotic-free DMEM/F12 culture medium at 37°C for 12 h, followed by washing with PBS and fixing with 4% paraformaldehyde. The fixed cells were washed with PBS, treated with DAPI followed by washing with PBS and visualized using fluorescent microscopy.

For visualization of cadherin-11 expression, the infected osteoblasts with *S*. *aureus* (non-FITC labeled) were fixed, blocked with blocking buffer (1% BSA containing 0.1% saponin in PBS) and treated with anti-osteoblast cadherin antibody (OBAb) in antibody dilution buffer (1% BSA, 0.1% saponin in PBS) and incubated at 4°C overnight. The cells were washed with PBS and treated with secondary Alexa Fluor 555 goat anti-mouse IgG in antibody dilution buffer for 2 h, treated with DAPI, washed and visualized under the fluorescent microscope.

To determine the binding and uptake of ACB-OBAb using microscopy, osteoblast cells (5 x 10^4^ cells) were cultured overnight on imaging dishes. The non-infected or infected osteoblasts (prepared as in intracellular antimicrobial assay) were treated with 1mL DMEM/F12 medium containing ACB-OBAb (10mg/L) and incubated at 37°C for 24 h. The osteoblasts were washed with PBS, fixed and treated with the blocking buffer followed by secondary Alexa fluor 555 goat anti-mouse IgG (against ACB-OBAb) in antibody dilution buffer. For infected osteoblasts, FITC rabbit anti-*S*. *aureus* IgG was also added to detect internalized *S*. *aureus* along with Alexa Fluor 555 goat anti-mouse IgG. The cells were incubated overnight at 4°C, stained with DAPI, and visualized employing fluorescent microscopy.

The antibacterial activity of ACB-OBAb on internalized *S*. *aureus* was determined using live/dead staining. Osteoblast cells (5 x 10^4^ cells) were seeded on D-lysine coated imaging dishes and cultured overnight. The osteoblast cells were infected with *S*. *aureus*, treated with gentamicin and washed with antibiotic-free DMEM/F12 culture medium, as mentioned above in the Intracellular antimicrobial assay. Following eradication of extracellular bacteria, the infected osteoblasts were treated with 5 mg/L of ACB-OBAb in 1mL of antibiotic-free DMEM/F12 culture medium and were incubated for 24 h. After incubation, the osteoblasts were washed 5 times with pre-warmed PBS to remove the extracellular *S*. *aureus*. The osteoblasts were then treated with 0.05% Saponin in PBS for 15 minat 37°C. The osteoblasts were washed with PBS and stained with the Live/Dead Stain (Invitrogen, Catalog # L-7012) that consists of two stains—Dye Syto9 (excitation 480nm/ emission 500nm) and Propidium iodide (excitation 535nm/ emission 617nm). The dyes were mixed in a 1:1 ratio, and 3μL of the dye mixture was added to each well-containing 1mL of PBS and incubated for 10 minin the dark. The imaging dishes were washed 3 times with pre-warmed PBS were visualized under a fluorescent microscope to determine the live and dead internalized *S*. *aureus*.

### MTT assay (cell viability)

Osteoblast cells (2 x 10^4^) were seeded on 96 well culture plates and cultured in DMEM/F12 for 48 h at 33.5°C followed by replacing with the culture medium with 200μL of varying concentrations of ACB-OBAb or ACB nanoparticles at 0 (control), 1, 2.5, 5, 10 and 20 mg/L and incubated for 48 h at 33.5°C. The effect of each treatment concentration was studied in a minimum of triplicates. After 48 h of nanoparticle treatment, the culture medium was replaced with 100μL of fresh culture medium followed by the addition of 10μL of 12mM MTT (3-(4,5-dimethylthiazol-2-yl)-2,5-diphenyltetrazolium bromide) to each well and incubation at 37°C for 4 h. 75μL of the culture medium was removed, followed by the addition of 50μL of DMSO and thorough mixing with the pipette. The cells were incubated at 37°C for 10 min, followed by measuring the absorbance at 540nm, and cell viability was presented as percentage of control.

### Statistical analysis

The results or data are presented as mean ± S.D. Graphpad Prism Software (Version 8.3) was used to conduct out the statistical analysis. Comparisons between two groups were carried out using Student’s t-test. A comparison between multiple groups was carried out using two-way ANOVA with Dunnett’s multiple comparison test. The significance threshold was set at 0.05 for the statistical analysis.

## Results

### Synthesis and characterisation of ACB and ACB-OBAb

**Fig 1:**
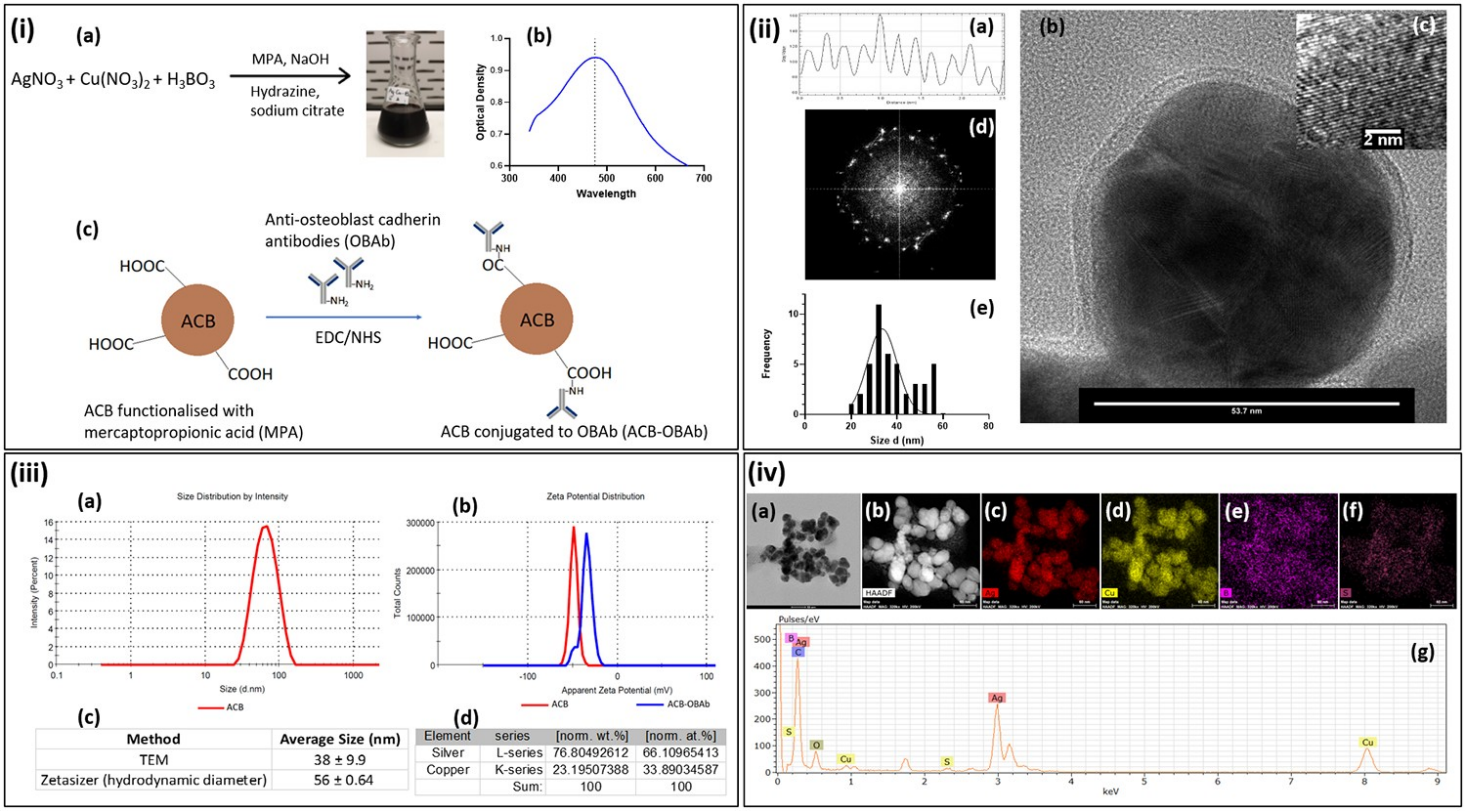
Characterization of nanoparticles. i(a) Schematic representation of the synthesis of ACB nanoparticles, i(b) Plasmon resonance of ACB nanoparticles and i(c) Schematic representation of the conjugation of ACB nanoparticles to anti-osteoblast cadherin antibodies. ii(a) Line scan for the interatomic distance from high-resolution TEM, ii(b) TEM image of a single ACB nanoparticle, ii(c) High-resolution TEM of ACB nanoparticles, ii(d) Fast Fourier Transform (FFT) image of the ACB nanoparticles and ii(e) Size distribution of the ACB nanoparticles. iii(a) Size distribution by intensity measured using dynamic light scattering, iii(b) Zeta potential measurement of ACB and ACB-OBAb, iii(c) Average size of ACB nanoparticles measured by TEM and Zetasizer and iii(d) Silver and copper percentage in ACB nanoparticles determined using Energy Dispersive X-ray Spectroscopy (EDS). iv(a) TEM image of ACB nanoparticles and ii(b) STEM image of ACB nanoparticles. iv(c), iv(d), iv(e) and iv(f) are the elemental mapping for silver, copper, boron, and sulfur, respectively. iv(g) EDS spectrum of ACB nanoparticles.

The tri-elemental silver-copper-boron (ACB) nanoparticles were synthesized using coprecipitation reaction, the schematic representation of which is shown in Fig 1ia. The plasmon resonance (Fig 1ib) of the alloy ACB nanoparticles had a maximum optical density at 476 nm, determining the formation of alloy nanoparticles. The OBAb antibody was conjugated to the ACB nanoparticles using EDC/NHS coupling (schematic representation, Fig 1ic). The line scan (Fig 1iia) obtained from High-Resolution TEM of ACB was used to determine the interatomic distance, which was found to be approximately 0.22 nm and could be typically due to alloy formation. The TEM image (Fig 1iib) of a single ACB demonstrated the formation of spherical nanoparticles. The High-Resolution TEM of ACB nanoparticle is given in Fig 1iic. Also, the Fast Fourier transform (FFT) image (Fig 1iid) of ACB demonstrated a crystalline structure. The size distribution from the TEM and the hydrodynamic diameter size distribution obtained from Zetasizer are shown in Fig 1iie and 1iiia, respectively. The zeta potential (Fig 1iiib) of the ACB and antibody conjugated ACB (ACB-OBAb) nanoparticles was determined to be -48.7 ± 5.96 mV and -34.6 ± 6.49 mV, respectively. The average size of ACB from TEM was found to be 38 ± 9.9 nm and its hydrodynamic size was found to be 56 ± 0.64 nm which are shown in Fig 1iiic. The EDS derived normalized atomic percentage (Fig 1iiid) of Ag and Cu in the ACB nanoparticles was found to be 2:1, respectively. The elemental mapping of the ACB nanoparticles utilizing Electron Dispersive X-ray Spectroscopy (EDS) was determined from the representative TEM (Fig 1iva) and STEM (Fig 1ivb) images which showed the mapping of silver (Fig 1ivc), copper (Fig 1ivd), boron (Fig 1ive) and sulfur (Fig 1ivf). The EDS spectrum (Fig 1ivg) shows the different elements present in ACB.

**Fig 2:**
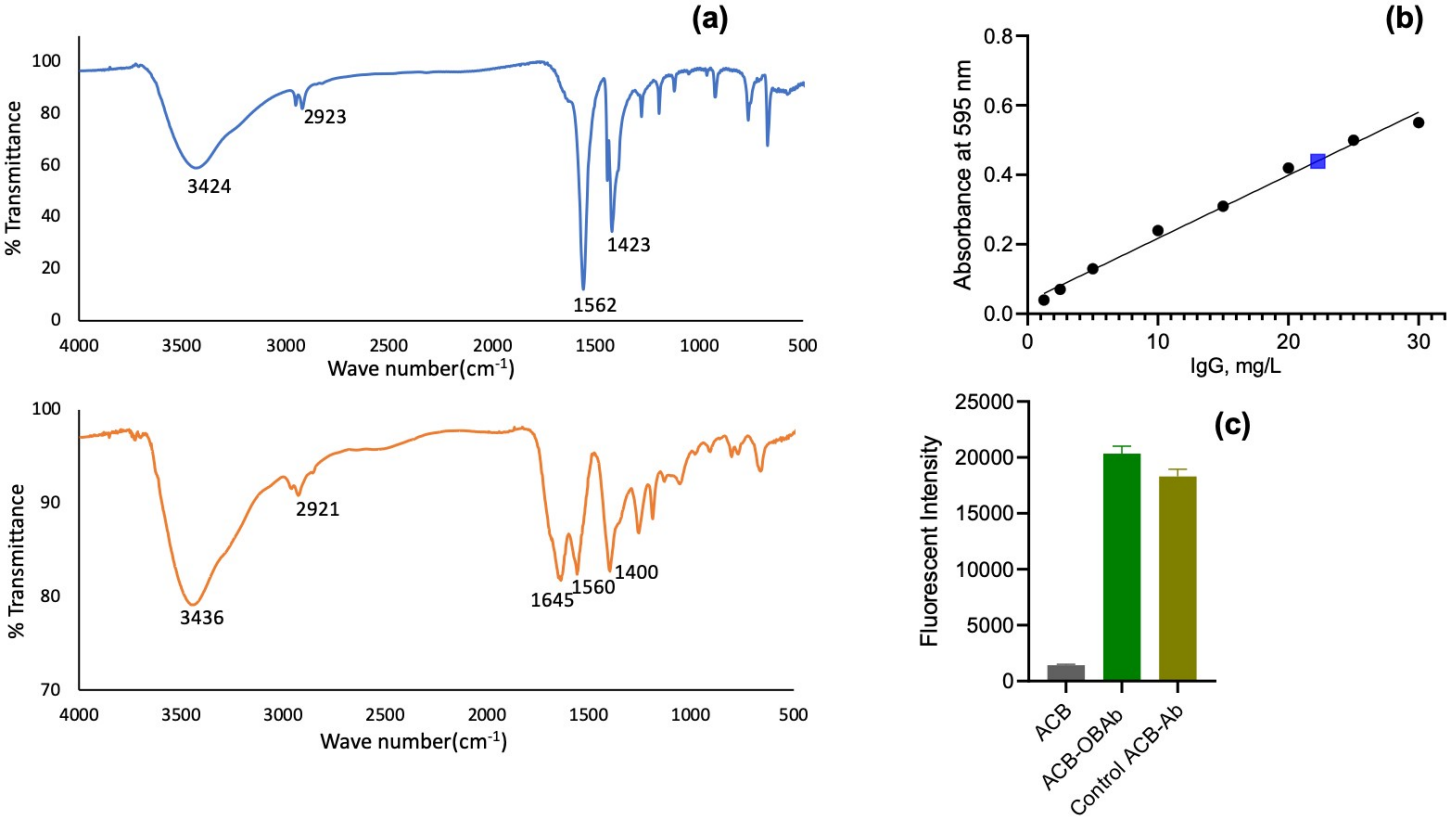
Characterization of conjugation of OBAb to nanoparticles. (a) Fourier Transform Infrared Spectroscopy (FTIR) of ACB and ACB-OBAb (Osteoblast cadherin antibody conjugated ACB nanoparticles), (b) Standard curve for IgG determined using Bradford assay. The blue marker represents the concentration of non-conjugated OBAb in the supernatant to determine the level of conjugation and (c) the fluorescent intensity of Alexa fluor 488 secondary antibody (goat anti-mouse IgG) bound to ACB-OBAb measured using spectrofluorimetry and the study was carried out in triplicates.

The conjugation of OBAb to ACB was also determined using FTIR (Fig 2a). The presence of a peak at 1645 cm^-1^ is indicative of an amide bond due to OBAb [64,65]. The amount of OBAb conjugated to ACB was determined using the Bradford assay. The standard curve of bovine IgG is given in Fig 2b. 50μg of OBAb was used for the conjugation of 1mg of ACB, and after the reaction, the supernatant contained 22μg of OBAb. It was determined that 28μg of OBAb was conjugated per mg of ACB.

### Antibacterial activity against extracellular *S*. *aureus*

**Fig 3:**
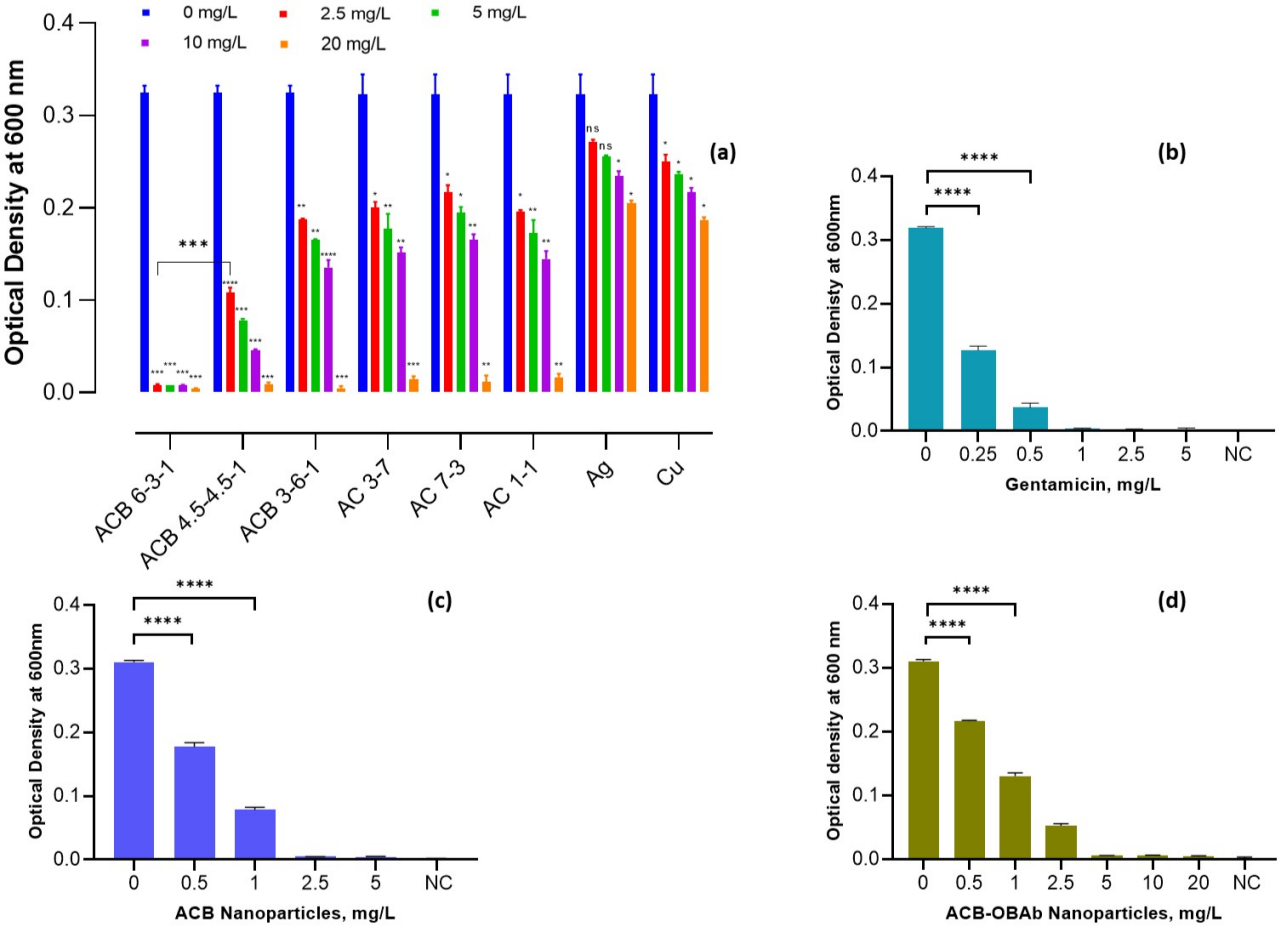
Antibacterial activity of nanoparticles and gentamicin against *S*. *aureus*. (a) Screening for the extracellular antibacterial activity of ACB nanoparticles (with varying ratios of Ag, Cu, and B). Each nanoparticle was tested in triplicates (n = 3). Statistical analysis by 2-way ANOVA with Dunnett’s multiple comparison test. Each bar represents the mean ± S.D. The statistical significance was compared to the non-treated (0 mg/L) group. **** represents P ≤0.0001, *** represents P ≤0.001, ** represents P ≤0.01, * represents P ≤0.05 and ns represents >0.05. A t-test was conducted between the antibacterial data sets of ACB (6-3-1) and ACB (4.5–4.5–1) at 2.5mg/L, which showed a statistical significance of P ≤0.001 (***). The significance threshold was set at 0.05 for the 2way ANOVA and t-test. (b) The antibacterial activity of gentamicin against extracellular *S*. *aureus*. (c) The antibacterial activity of ACB nanoparticles against extracellular *S*. *aureus*. (d) The antibacterial activity of ACB-OBAb nanoparticles against *S*. *aureus*. For images (b), (c), and (d), each bar represents the mean ± S.D. The statistical significance was compared to the non-treated group using a t-test. The significance threshold was set at 0.05 for the t-test.

The antimicrobial activity of the synthesized nanoparticles was conducted by treating *S*. *aureus* with different concentrations (mg/L) of metallic nanoparticles in DMEM/F12 medium and measuring the optical density at 600nm. Antibacterial screening for the effect of the composition of the metallic nanoparticles by varying the concentration Ag, Cu and B was carried out. It was found that the antibacterial effect increased in the following order: Tri-elemental Ag-Cu-B (ACB) > Bimetallic Ag-Cu (AC) > monometallic nanoparticles Ag or Cu. The presence of boron and the ratio of Ag to Cu affected the antibacterial activity. The tri-elemental Ag-Cu-B (ACB 6-3-1) with the percentage of Ag, Cu and B at 60%, 30% and 10% respectively exhibited the highest antibacterial effect in DMEM/F12 culture medium (Fig 3a). The minimum inhibition concentration (MIC) was determined for the ACB, ACB-OBAb and gentamicin. The MIC is the minimum concentration of the antimicrobial agent, which does not show any significant increase in optical density at 600nm after 6 h of incubation. The MIC for ACB, ACB-OBAb and gentamicin was found to be 2.5 mg/L, 5 mg/L and 1 mg/L, respectively.

### Cadherin-11 expression and ACB-OBAb targeting using fluorescence microscopy

**Fig 4:**
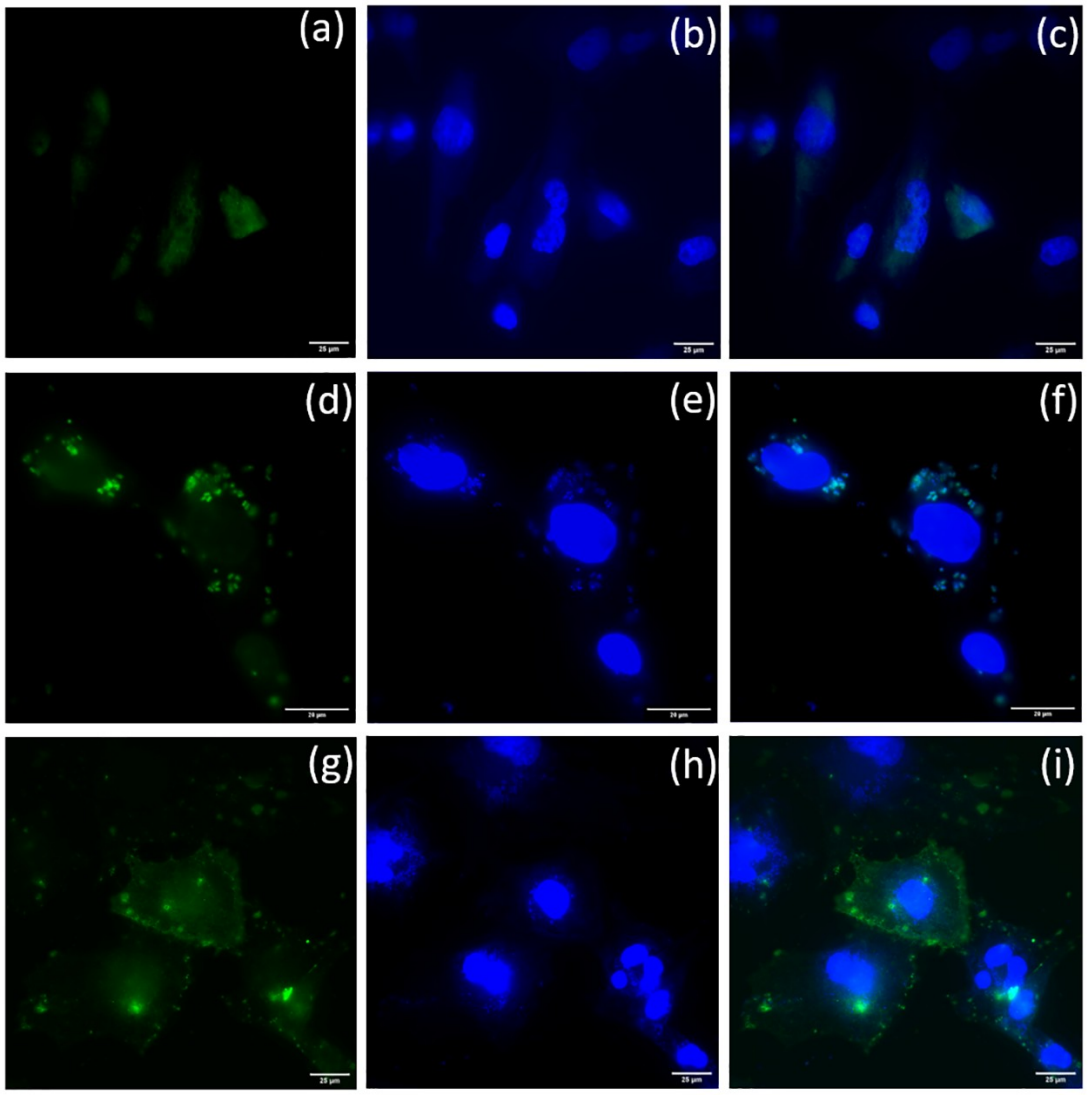
*S*. *aureus* internalization and cadherin expression. Upper panel (scale 25μm)—Noninfected osteoblasts. (a) Osteoblasts exhibiting autofluorescence. (b) Osteoblasts stained with DAPI (blue) and (c) Merged image of the non-infected osteoblasts. Middle panel (scale 20μm)–*S*. *aureus* infected osteoblasts. The *S*. *aureus* bacteria were labeled with FITC (green). (d) Osteoblasts with internalized *S. aureus* appear green, indicating internalization. (e) The infected osteoblasts are stained with DAPI (blue). Osteoblasts nucleus as well as the *S*. *aureus* (small blue spots) were stained with DAPI, which appear blue (f) Merged image of the infected osteoblasts. Lower Panel (scale 25μm)–Cadherin-11 expression by infected osteoblasts. Osteoblasts were treated with primary mouse anti-osteoblast cadherin antibodies and counterstained using Alexa fluor 555 (AF555) labeled secondary goat anti-mouse IgG. (g) Osteoblast expressing cadherin-11 labeled with AF555 secondary antibody (green). (h) Osteoblasts labeled with DAPI (blue). (i) Merged image of g and h.

**Fig 5:**
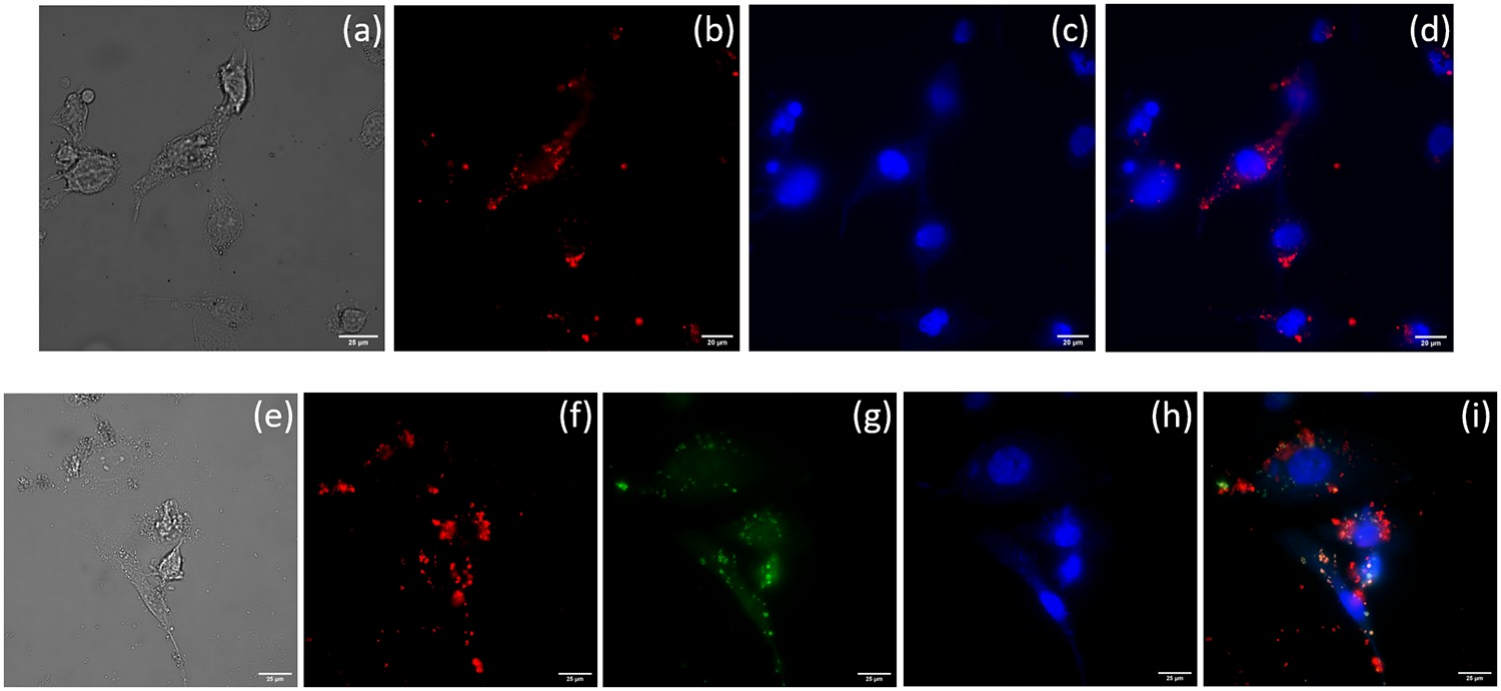
Internalization of ACB-OBAb by osteoblasts. Upper Panel (scale 20μm)—The binding of ACB-OBAb to osteoblasts. (a) DIC image of osteoblast treated with ACB-OBAb. (b) ACB-OBAb stained (red) with AF555 secondary goat anti-mouse IgG. (c) The ACB-OBAb treated osteoblasts treated with DAPI (blue). (d) Merged image of (b) and (c) showing the binding of ACB-OBAb (red) binding to osteoblast. Lower Panel (scale 25μm)—The binding of ACB-OBAb to infected osteoblasts. (e) DIC image of osteoblast treated with ACB-OBAb. (f) ACB-OBAb stained (red) with AF555 secondary goat anti-mouse IgG. (g) The internalized *S*. *aureus* stained using FITC labeled anti-*S*. *aureus* mouse IgG. (h) The infected osteoblasts treated with DAPI (blue). The *S*. *aureus* was also stained with DAPI besides the osteoblast nucleus. (i) Merged image of f, g, and h, showing the binding of ACB-OBAb to infected osteoblasts.

The internalization of the FITC labeled *S*. *aureus* by osteoblasts was confirmed using fluorescence microscopy, as shown in Fig 4(d), 4(e) and 4(f). The Cadherin-11 expression was confirmed by fluorescence microscopy, as shown in Fig 4(g), 4(h) and 4(i). The targeting of ACB-OBAb to non-infected osteoblasts is represented by the Fig 5(a)–5(d). The lower panel of Fig 5(e)–5(i) represents the targeting of ACB-OBAb to infected osteoblasts. (S1 Fig. The binding of the ACB-OBAb to non-infected osteoblasts was shown by employing an AF-594 dye-labeled goat anti-mouse IgG targeting the primary OBAb antibody).

### Intracellular antimicrobial activity of ACB and ACB-OBAb

**Fig 6:**
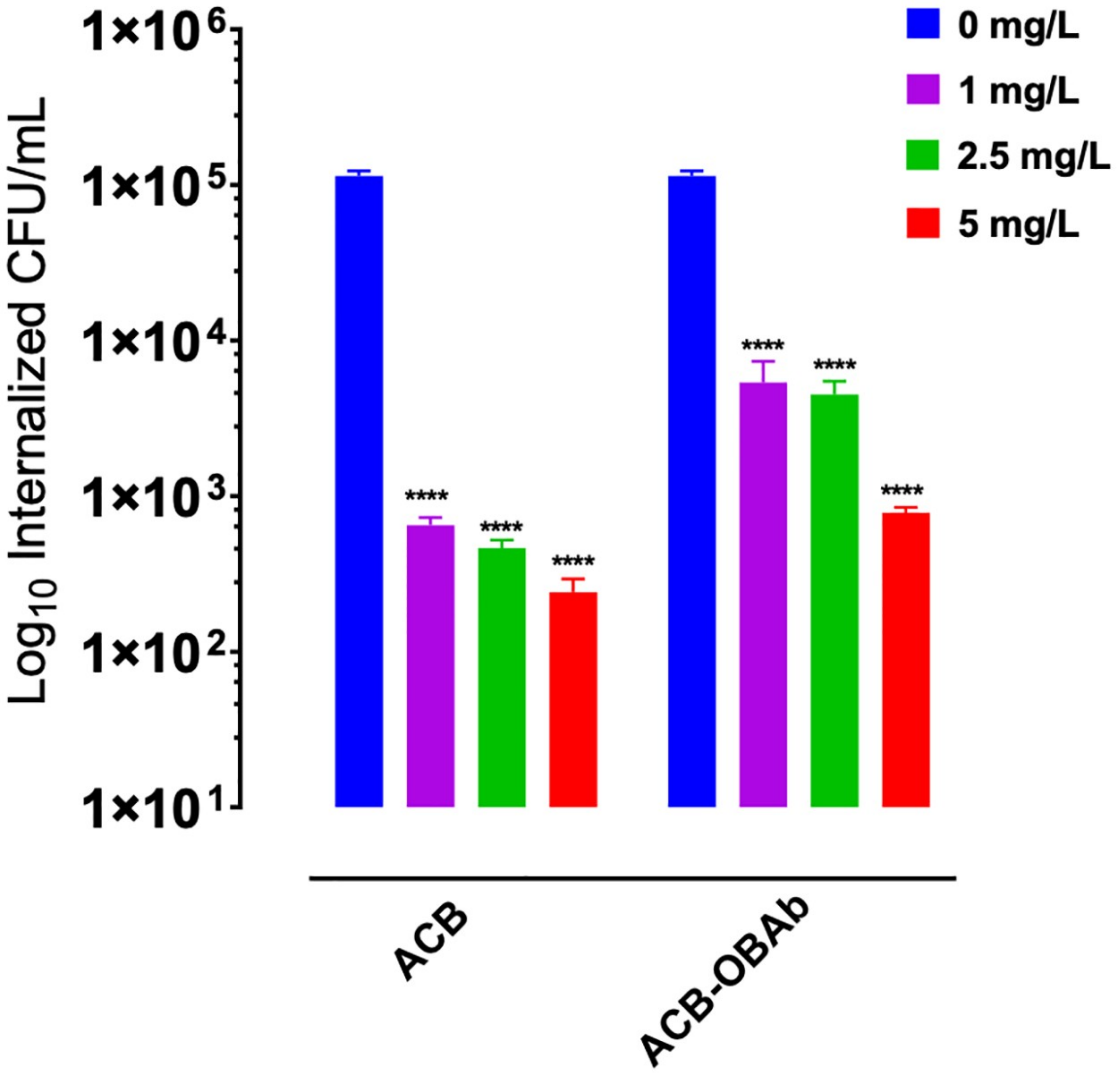
The antibacterial activity of ACB and ACB-OBAb against internalized *S*. *aureus*. Each bar represents the mean ± S.D. Each nanoparticle treatment concentration against internalized bacteria was tested in a minimum of triplicates. The statistical significance was compared to non-treated (0μg/mL) group) using a t-test. **** represents P-value ≤0.0001. The significance threshold was set at 0.05 for the t-test.

The antibacterial activity of ACB and ACB-OBAb against intracellular *S*. *aureus* in osteoblasts is shown in Fig 6. There was 2.2 log and 2.67 log decrease in internalized bacterial load for the infected osteoblasts that were treated with 1 mg/L and 5 mg/L of ACB nanoparticles, respectively, when compared to the control (0 mg/L). However, for the targeting ACB-OBAb nanoparticles, there was a 1.32 log and 2.16 log decrease in bacterial load at 1 mg/L and 5 mg/L nanoparticle concentration, respectively. The difference in antibacterial activity between ACB and ACB-OBAb could be due to the targeting nature of ACB-OBAb and the proportionality of cadherin expression by osteoblasts. Both ACB and ACB-OBAb did not show any CFU formation or growth at a concentration of 10mg/L (not shown). (S2 Fig shows the 24 h treatment of internalized bacteria with gentamicin which did not show any reduction in bacterial load when compared to the control).

### LIVE/DEAD staining of nanoparticle treated internalized *S*. *aureus*

**Fig 7:**
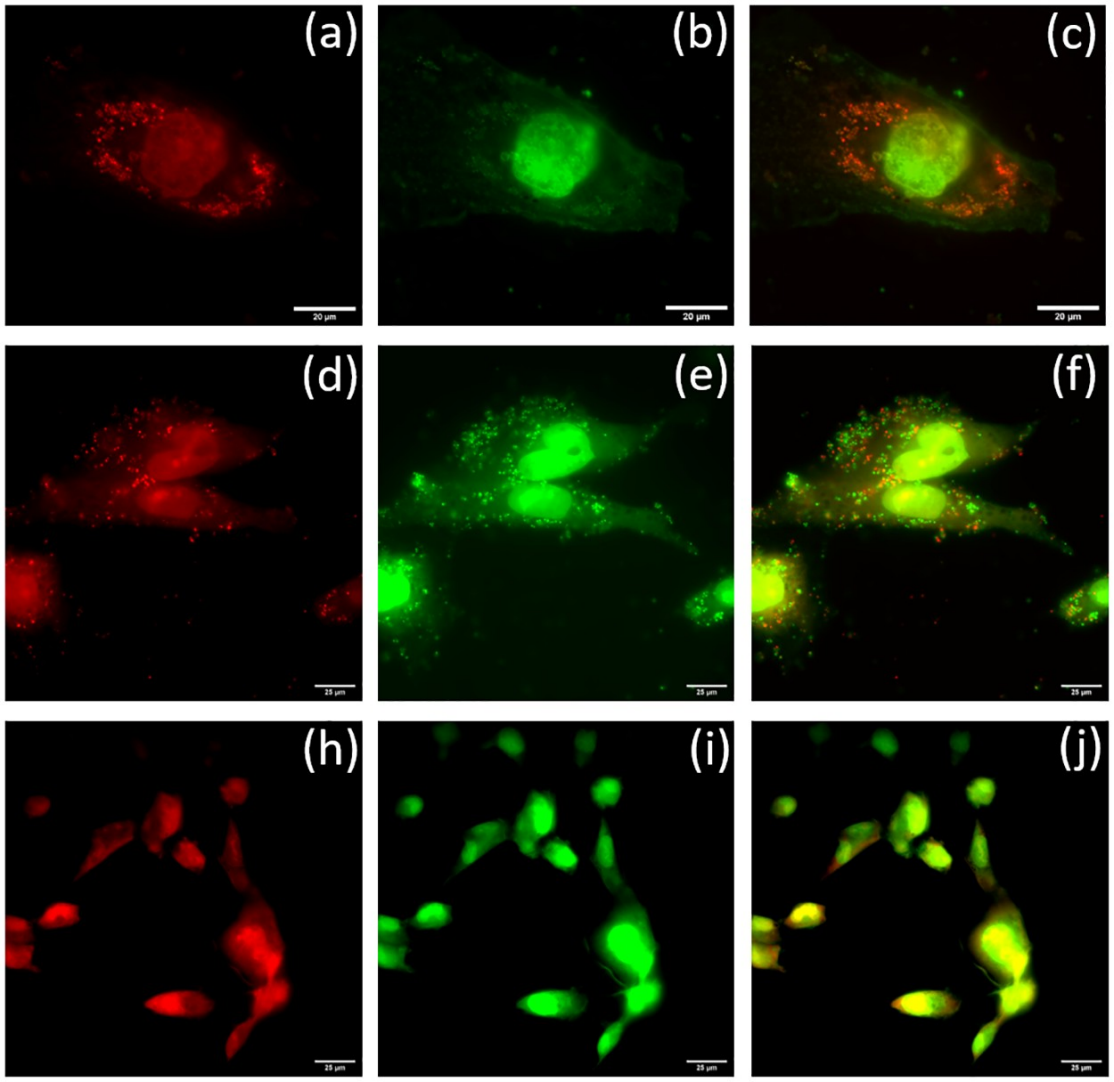
Live/dead bacterial staining of infected osteoblasts. Propidium iodide (PI) (Ex 535/ Em 617) Syto 9 (Ex 485/ Em 498) Staining. Upper panel (Scale 20μm): *S*. *aureus* infected osteoblast treated with 5mg/L of ACB-OBAb. (a) PI (red) staining showed dead intracellular *S*. *aureus* (small red spots). PI is not permeable to live cells. (b) Syto 9 (green) staining which is taken up by both live and dead bacteria. (c) Merged image in which the orange-red spots indicate dead bacteria. Middle Panel (Scale 25μm): Infected osteoblasts not treated with ACB-OBAb. (d) PI staining—small red spots indicate dead bacteria, (e) Syto 9 staining in which the green spots indicate live bacteria. (f) Merged image of (d) and (e). A higher number of live bacteria was seen in non-treated infected osteoblasts. Lower Panel (Scale 25μm)–Control: Non-infected and not treated with ACB-OBAb. (h) PI stained osteoblasts. (i) Syto 9 stained osteoblasts. (h) Merged image of (h) and (i), which does not show green or red spots of dead or live bacteria.

The live/dead staining of *S*. *aureus* in infected osteoblasts treated with 5mg/L of ACB-OBAb is shown in Fig 7(a), 7(b) and 7(c), which indicates a significant number of dead bacteria due to nanoparticle treatment. The Fig 7(d), 7(e) and 7(f) show the live/dead staining of infected osteoblasts that are not treated with ACB-OBAb and indicate the presence of a high number of live bacteria when compared to the treated infected osteoblasts. The Fig 7(g), 7(h) and 7(i) are the control non-infected osteoblasts that do not indicate any bacterial presence when compared to the infected osteoblasts. (S3 Fig represents the live/dead staining of internalized *S*. *aureus* treated with 2.5mg/L of ACB-OBAb and the control which are the infected osteoblasts not treated with ACB-OBAb).

### MTT assay

**Fig 8:**
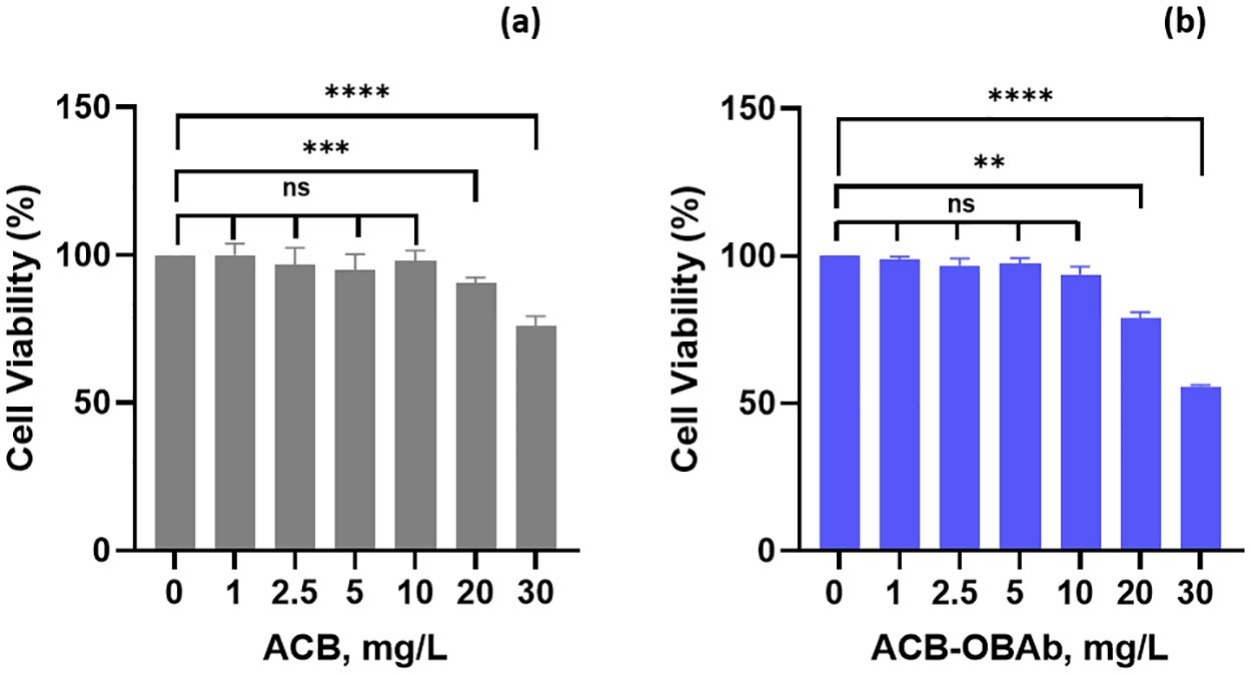
MTT assay. The cytotoxicity of (a) ACB to osteoblasts (b) ACB-OBAb to osteoblasts. Each bar represents the mean ± S.D. The cytotoxicity of each nanoparticle concentration to osteoblast was carried out in a minimum of triplicates. Each treatment concentration was compared to the control (0 mg/L) using a t-test with Welch’s correction. **** represents P-value ≤0.0001, *** represents P-value ≤0.001, ** represents P-value ≤0.01 and “ns” represents non-significant. The significance threshold was set at 0.05 for the t-test.

The MTT assay helps to the viability of osteoblasts that were cultured with different concentrations (0 to 30mg/L) of ACB and ACB-OBAb. The osteoblasts showed good viability for both ACB (Fig 8a) and ACB-OBAb (Fig 8b) up to 10mg/L concentrations after 48 h of incubation. The osteoblast viability decreased to 90% and 78% after treatment with 20mg/L of ACB and ACB-OBAb, respectively. The viability was further reduced to 75% and 55%, when treated with ACB and ACB-OBAb, respectively, at 30 mg/L concentration of nanoparticles.

## Discussion

Ag and Cu nanoparticles are efficient antibacterial agents against extracellular as well as internalized *S*. *aureus* as the Ag^+^, or Cu^2+^ ions can penetrate the mammalian cells [38,39]. Also, the alloyed nanoparticles of silver and copper possess higher antibacterial activity than mono-metallic silver or copper nanoparticles[29,35–37]. The increase in antibiotic resistance and the non-permeability of antibiotics to mammalian cells are some of the problems faced by current therapeutic strategies[17,18]. Hence alloyed metallic nanoparticles may become potential candidates for alternative therapy. The current challenges related to the use of metallic nanoparticles is their toxicity and non-specific accumulation in different organs that were reported from animal studies[53,54]. Our approach to reducing the toxicity is by targeting the metallic nanoparticles to the site of infection by conjugating the nanoparticles to targeting biomolecules.

In this study, tri-elemental ACB (Ag 60%, Cu 30% and B 10%) nanoparticles exhibited the highest extracellular antibacterial activity (Fig 3a and 3c) when compared to bimetallic (Ag-Cu) or monometallic (Ag or Cu) nanoparticles. The addition of a metalloid like boron to the Ag-Cu nanoparticle structure significantly increased the antimicrobial property. The ratio of silver and copper in the trielemental ACB also affected the antibacterial efficacy of the nanoparticles. The elemental analysis of the nanoparticles confirmed the presence of silver, copper, boron and sulfur (Fig 1iv). Sulfur was detected due to the mercaptopropionic acid used for capping ACB nanoparticles. FTIR and fluorescent spectrophotometry confirmed the conjugation of OBAb to ACB. The MIC of ACB-OBAb was found to be 5mg/L (Fig 3d) which is slightly higher than the non-conjugated ACB (MIC = 2.5mg/L, Fig 3c) for the extracellular *S*. *aureus*. For the gentamicin control, the MIC was found to be 1mg/L (Fig 3b) which was better compared to the metallic nanoparticles(Fig 3b); however, gentamicin is not effective against intracellular infection, when compared to metallic nanoparticles.

Non-alloy, either Ag alone or Cu alone nanoparticles, are less effective than alloy Ag-Cu nanoparticles. The synergistic effect of Ag and Cu may enhance the antibacterial activity of Ag-Cu nanoparticles [29,35–37]. Silver ions induce reactive oxygen species that create alterations in the bacterial compounds and also, silver ions bind to sulfur and oxygen of bacterial molecules, thereby inhibiting bacterial growth. Copper ions induce hydroxyl radicals that damage bacterial proteins and DNA [36]. Boron nanoparticles have been shown to be antibacterial [66] and boron has an anticorrosive effect on metals [51,52]. In our previous studies, it was shown that Boron doped Ag-Cu nanoparticles were effective against both extracellular and intracellular *S*. *aureus* infection [38,53]. The combined antimicrobial effect of the elements Ag, Cu and B along with the controlled release of metallic ions due to anticorrosive boron may enhance the overall effect of trielemental ACB nanoparticles.

Cadherin-11 is the most expressed cadherin in human osteoblasts [62,63]. The OBAb antibodies conjugated to ACB target the extracellular domain of Cadherin-11 (Fig 4i) in osteoblast cells. Incubation of ACB-OBAb nanoparticles with infected osteoblasts resulted in the binding and internalization of the nanoparticles (Fig 5i). This demonstrated that our ACB-OBAb nanoparticles were able to target the Cadherin-11 expressing infected osteoblasts. This method of targeting could be used as a potential strategy to target infected osteoblasts by employing specific biomarkers that are expressed due to infection. Both the non-conjugated and ACB-OBAb nanoparticles were able to reduce the bacterial load of infected osteoblasts after 24 h of infection. Non-conjugated ACB nanoparticles resulted in 2.24 log reduction, whereas ACB-OBAb nanoparticles caused 1.32 log reduction of *S*. *aureus* at a treatment concentration of 1mg/L (Fig 6) and the difference in antimicrobial activity may be due to the ACB-OBAb internalization which is proportional to the expression of Cadherin-11. This receptor-based internalization of nanoparticles depicts that overexpression of disease-based receptors will cause more internalization of nanoparticles and hence will enhance the therapeutic efficiency.

To make sure that the reduction in infection observed is solely by the effect of nanoparticles and not due to the killing of the infected osteoblast cells, the MTT viability assay did not show toxicity to osteoblasts at doses ≥20mg/L after 48 h of incubation. This can be compared with the biocompatibility of silver based nanoparticles that showed lower toxicity to mammalian cells that were tested using MTT assay [43,67]. Hence the reduction in infection is due to the targeted nanoparticles *via* cadherin-11. The ACB-OBAb nanoparticles showed higher toxicity to osteoblasts at 20mg/L and 30mg/L when compared to non-conjugated ACB. This could be due to the targeting of ACB-OBAb to osteoblasts, which results in the accumulation of the nanotherapeutics in the infected cells or at the infected site [68,69]. However, the intracellular ACB-OBAb showed a significant reduction in intracellular osteoblast infection at 1mg/L (1.32 log reduction), which is 20 times lesser than the toxic dose we found against osteoblasts.

In summary, the composition of the nanoparticles played an essential role in the antibacterial activity. ACB alloy nanoparticles were more effective than bimetallic alloyed (Ag-Cu) or monometallic (Ag or Cu) nanoparticles. This study demonstrated that ACB-OBAb nanoparticles were able to target the osteoblasts and significantly reduce the internalized infection *in vitro* and paves the way for future studies in animal models utilizing specific targeting strategy whereby a specific biomarker expressed in infected osteoblasts could be targeted to increase targeted delivery of therapeutic levels of nanoparticles.

## Supporting information

S1 Raw data (XLSX)

S1 Fig (PNG)

S2 Fig (PNG)

S3 Fig (PNG)

S4 Fig (ZIP)

S5 Fig (ZIP)

S6 Fig (ZIP)
