# Supplementary figures and images for: Boron doped silver-copper alloy nanoparticle targeting intracellular S. aureus in bone cells
Source: PLoS One. 2020 Apr 10;15(4):e0231276. doi: 10.1371/journal.pone.0231276 (PMC7147743; doi:10.1371/journal.pone.0231276)

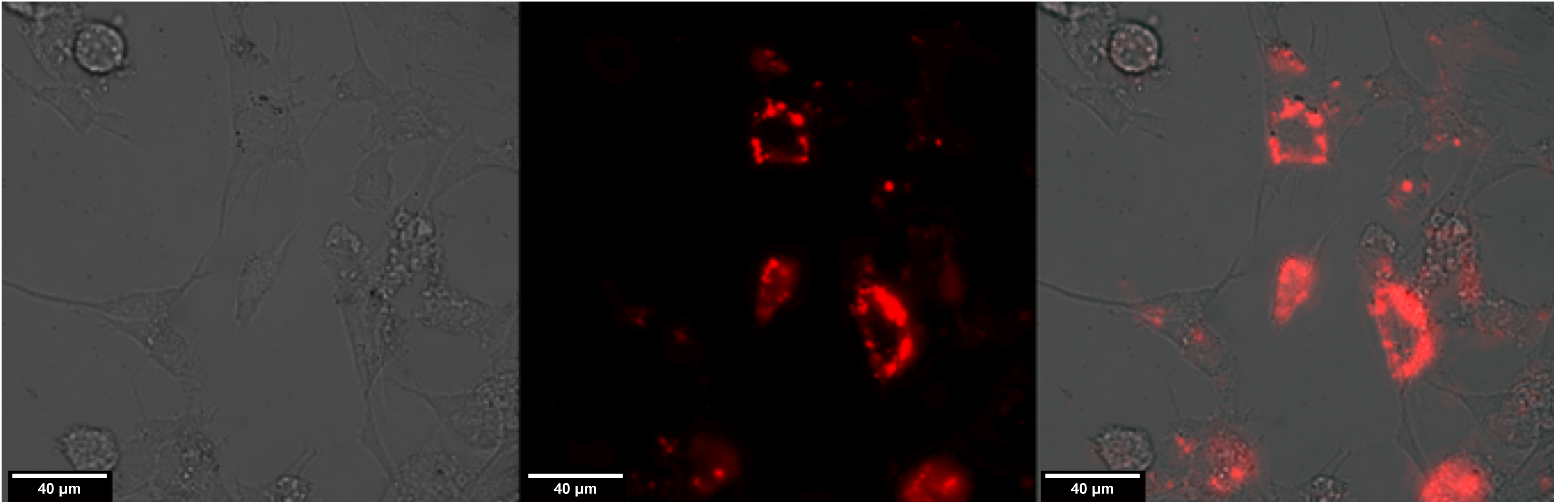

Supplement: S1 Fig — (PNG) [file pone.0231276.s002.png]

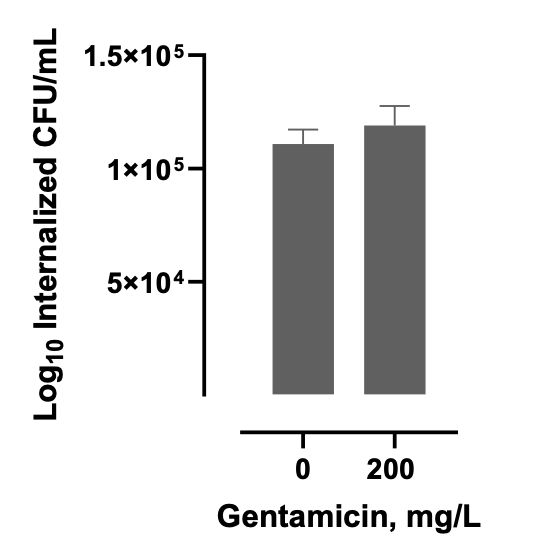

Supplement: S2 Fig — (PNG) [file pone.0231276.s003.png]

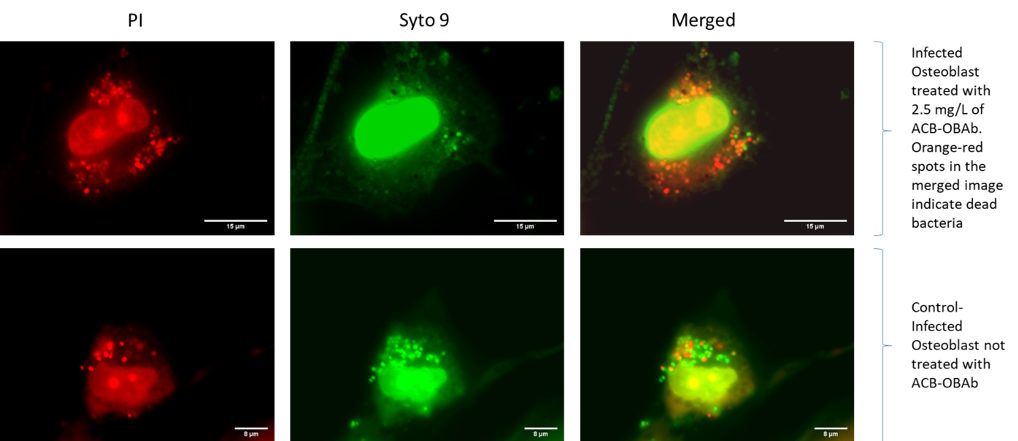

Supplement: S3 Fig — (PNG) [file pone.0231276.s004.png]
